# A Plea for Dignified Dental Fees

**Published:** 1906-01

**Authors:** T. Ledyard Smith

**Affiliations:** New York, N. Y.


### A PLEA FOR DIGNIFIED DENTAL FEES.

By Dr. T. Ledyard Smith, New York, N. Y.

The average dentist does not toil day by day with the ultimate view of having his name erected in the Hall of Fame. And when he dies—well, we read obituary notices of clergymen, lawyers, physicians, actors, actresses, merchants, politicians and so on; but a notice in any prominent newspaper, other than some small local sheet, of the death of a dentist, is a rarity. He is not known to the public. The newspapers never heard of him. The people do not say: "Well, well! That's too bad. Dr. Bridge is dead." His death is a loss to a few who have grown to like the interest he had taken in the welfare of their mouth and teeth. Still, there are dentists who live and work with the hope of gaining this past announcement. But the average dentist would change his occupation for a more remunerative one if he could be assured with a certainty that the other would yield a larger return.

Young men of wealth do not engage in dentistry. The man who graduates hustles to even pay rent, and for the most part a cheap one at that. To meet this he must build business. To induce patronage he establishes a low fee, and oftentimes executes clever, painstaking operations for labor union wages, where, in trades, knowledge and superiority of one man's ability over another counts for naught.

It is quite safe to say that the training a dentist must have is an unknown factor to the majority of people. To them he can pull teeth, fill teeth and make plates and bridges, something which they believe can be learned by anyone in a short time. Any enlargement of this scope is news to them. The range of knowledge required in dentistry in its every department is entirely foreign to the mass of people. They know that you can make a bridge; but that it requires more than porcelain teeth and gold (a low K grade at that) to perfect a bridge, or that more than a tinman's knowledge is required is all news to them. It is so with every department in dentistry. The magnitude of the commercial side, with its invested fortunes; the trade industry, with its thousands of skilled workers, with the enormous effort toward finished aids to the dentist, this is as much undreamed of as is the extent of the art of dentistry itself, with all its requirements. The scope covered by the dentist is unknown. The dentists's training, the time required for the first years of study, never thought of. The value of the great benefits to be had are unappreciated. The esteem in which dentistry is held is in exact proportion to the knowledge possessed by the masses, and which they in no better way make known than by their unwillingness to pay bills that may be in excess of some small limit set by themselves.

In the main, dental bills have always been considered too large. In nearly every instance they are at least fifty per cent. less than they should be. Dental fees, no matter from whom received or to whom paid, or for what service rendered, are, if considered with regard to all relative conditions, too small in amount. Look at it in any way you will, the argument is in favor of higher fees. The service rendered—no matter what— is of more real value to the patient than is paid for, considered from the standpoint of comfort from an unhealthful or foul condition; freedom from a condition that primarily may affect the stomach, and secondarily the system of assimilation, converting contents into a condition that may be expressed in disorders that could be traced to blood burdened with and carrying impurities.

The freedom from any oral pain is worth more than any fee that is ever paid for its relief.

Many people expect greater durability in dental operations than they find in nature. Unless a dentist is infallible they imagine their money ill-spent.

A tooth that is saved to its owner for a period of even two or five years is worth more than any fee ever charged against it, whether that fee be one dollar to some poor person or one hundred dollars to someone who is rich. The sum paid is insignificant to the real value received as compared with other expenditures they make, where the return is in many instances trifling and of no real or lasting benefit. Every dental operation of any character whatever is paid for only in a meagre degree to the value given.

Where the hideous disfigurement of being without a tooth is changed by imitating with dental art the color, form and harmony of nature, this operation alone should be and in reality is worth many times more than the bill rendered for such work. If persons can have such operations done for nothing, why of course they are fortunate. If it were impossible to have this done for less than fifty or even one hundred dollars, they would still be fortunate, for who can value this sum more than an unsightly break in a row of otherwise well appearing teeth? For any kind of porcelain crown that gives promise to endure with satisfaction for a period of over one year, the price of, we will say, ten dollars, is an insult to the knowledge that every dentist must possess. It belittles the very institution of dentistry.

The wide range of porcelain inlay work, with its great possibilities, its immunity from degenerating influences, together with its beauty, this, too, is destined to fall in the mire of cheap prices. Dentists say no. But this is because those dentists consider ten or twenty dollars high for an artistic inlay, whereas no inlay work should be performed for any such paltry sum. If the dentist undervalues the mechanical skill, the beauty and the art surrounding inlay work, naturally, the patient will share his estimate. The first lesson to the patient toward a correct understanding of porcelain work should be its price. But these things are otherwise. It is positively saddening to see this beautiful field plebeianized by cheap prices. And if it commences thus, what excuse can be offered to quadruple its cost? What hope can be had where the whole domain of dental usefulness and dental art is cheapened and belittled by the very ones who, in order to make a living, set this present low standard?

Neither figures nor words are broad enough to express the comforts and health-extending benefits derived from dentistry. The pity is for the estimate placed on its wide field of importance by the very ones who should place its standard above that of a union trade.

There should be a universal activity among dentists toward higher fees. A general tendency to even double the present fees, whatever they may be. A general harmonious effort and movement should be made in this direction. An effort to act in concert. The whole profession to act as an organization. The people should be checked in their pleasure of shopping for dental prices. There should be no bargains in dentistry. There are dentists who will draw their faces into a look of importance, and with scorn tell you that they are not troubled with shoppers. True, but rich as their patrons are, should a bill of one hundred dollars be rendered for a single tooth, or several hundred for several teeth, there is a chance of a protest, if not discontinuance of patronage. And yet that same person without any hesitancy will renew tires on his automobile once or twice a month at fifty dollars a pair. His tips to ignorant waiters, barbers, pages, menials in general, will run well into several hundred dollars a year, and in ten years he will have paid these ignorants many times over any sum he will have paid out for dentistry, where art, skill, learning and knowledge is its very foundation. The menial he so willingly tips is the very antipode of the dentist, to whom he will begrudge a just fee. Try him on a good liberal fee and see him wince.

Any person regardless of station, spends more in ten years on barber tips, waiter tips and similar gratuities than he does on dentistry. This low standard of public education lies at the door of dentists. Their low fees have taught a depreciation of the importance of dental services.

We read much about having the educational standard of dentistry raised for those who wish to enter; but after twenty-five years I see but little evidence that there is any educational elevation in the minds of the people in regard to dentistry. I see no advance in their knowledge of dental matters over what they displayed years and years ago. That dentistry has progressed, yes. That the education of the people has, no. There is this difference—they expect more today for one hundred cents than they once did.

Remove dentists, and you have a homely, grimace-looking population constantly tortured with distracting facial aches, followed by a train of ills that are the sequential expression of disordered mouths.

In addition to the injury which the profession does itself by charging low fees, as if not content with giving their services for half what they should bring, it commits another error, and in a measure a worse one, in adhering to the pernicious credit system. The aim of dentists toward stupid professionalism and ethics that lack common sense, has brought about an understanding in the minds of people to the end that they are insulted if a dentist insists that he is working for money—cash. The people have been unwisely encouraged to believe in this foolish and injurious method of working for them without any mention before or after work of the cost of their obligation. The result is—but, I am talking to dentists, and every dentist with deep regret knows the result—a long list of accounts that represent on his part thought, time, actual labor, nerve-straining tension, skill, art, together with one, two, three or more dollars a day out (whatever the average may be) for dental supplies—for all this, he is stupidly willing to let that person, if a stranger, escape without paying; if a regular patron, wait until a bill is sent, then another, then a polite letter, and so on, giving time credit that would bankrupt any commercial business, and all for what reason? For no sensible reason whatever. The credit system is destructive, pernicious in the extreme, without one excuse for its maintenance.

There is hardly a dentist who is even willing to acknowledge to himself the money he has lost in bad accounts in, say, ten or twenty years—money for which he has conscientiously worked hard. He has hundreds out that he would discount at fifty per cent. A portion of that he would let go at ten per cent, and a big percentage for a certainty will always remain a loss. This comes from a silly idea to maintain an office on some plan other than a common sense business one. The result is the price of stupidity.

However, the dentist gets along, and tries to live at least like a gentleman. That is something. But he never makes enough to be pointed out as a wealthy citizen. He is never fashionable. He gives no functions. He never entertains above four covers. His yacht never grows above a half-rater. His auto extravagance is confined to those in the hundreds—one in the thousands would bankrupt him. He frequents places where ten cents is the limit tip. He lives all through life second-class. He gets this for his learning, his knowledge, his skill, his art. He has been a public benefactor.

If at fifty or sixty, when his life is nearly spent, when he is gray and sour, if at that time he has a little money, he has saved it by having denied himself all the hundred and one enjoyments that are dear to all our people; things the enjoyment of which can only be had and appreciated by the young, each five years in one's life graduating one from certain pleasures, and bringing newer and fewer ones, until at sixty but few desires remain. At that period there is the ever near possibility of crape hanging at his door, and when it does a few people will think of how he hurt them once with a clamp or perhaps while adjusting a crown, and they will regret the "fortune" they spent on their teeth. That he made homely children better looking; that he preserved the teeth and the health of the middle-aged; gave useful substitutes to others, or that he was the one person to whom one goes early after a night of maddening pain—these things will have escaped their memory.

He may at least die with the consciousness and satisfaction of having lived a life of usefulness, having contributed his full share of effort to the generation in which he lived, but in which he was only a dentist.—*Items of Interest.*

Dr. C. S. Stockton, of Newark.—Now suppose for instance my good friend Dr. Jarvie can do in an hour what he conscientiously charges ten dollars for. That same patient comes to me, and I do an equal amount of work, and do it equally as skillfully, but I take two hours to do it. There is no question about the quality of the work. What is the result? Nine out of ten of the patients will say that Dr. Jarvie is an extravagant charger—almost a robber—and that Dr. Stockton is a moderate charger. We have done the same amount of work exactly, and have done it equally well. I say here tonight before you that Dr. Jarvie should have been paid more than I was paid, because that patient was freed for an hour from the unpleasantness of the dental chair. If that patient were a broker, doing business in Wall Street, he could go there and make perhaps five hundred or a thousand dollars in that hour that I had deprived him of, and that Dr. Jarvie had given him. That is a question worth thinking about.

Now after what I have said, let me ask you something else: What are you going to do with the fees you receive for your work? I speak of it because of experience—one of the best teachers there is. I commenced very young in practice, and had a little money. If I had continued as I started out, when I got a hundred dollars, to invest it in some good security—I would have been worth to-day a quarter of a million dollars or more. Instead of that, I thought I knew more than the average man. I loaned money, and took risks for a large amount, and to get my money back (the party had failed) I took the factory and thought I could run it in connection with my business. I could not be in two places at the one time. I spoiled for the time being my practice, and the factory ruined me. That is why I say to you, young men, when you get a hundred dollars, put it in something that is established, some good bank or railroad stock or bond and keep it. You are charging fees not for your present living, but to have something when you are old that you can rely on, and keep you in comfort.

Dr. B. F. Luckey.—What is the proper basis for professional fees? That question is quickly and easily answered, aside from all charitable work which every dentist is for one reason or another obliged to do—an equal charge for equal work for *all* patients. We have no moral right to punish one person because he has means, or in other words to charge the rich that the poor may pay less. It is unfair both to the rich and the operator. The poor can find in every city competent operators who are willing and able to work conscientiously for a fee that the poor can pay. The Government does not charge the rich three cents for a postage stamp, that it may be able to sell one to the poor for one cent. All people applying to the ticket office of a railroad are compelled to pay the same price for a ticket to a given point. It is a simple business proposition, and every well regulated dental office ought to be conducted on a business basis.

Fees should be based upon the ability to keep each day fully occupied. If by charging ten dollars per hour I can fill every hour each day, I am justified in charging it and am not exorbitant; but if I can only fill two or three hours each day at such fee, and could fill all at five dollars an hour, then my fee would be exorbitant. Many men can fill up every day on a basis of two dollars an hour who could not fill one day a month on a basis of ten dollars an hour. Each man must be a law unto himself, being governed by his environment in the beginning, and later by the environment that he is able to create.

Now, as to the first question. Why do people who sometimes think I am a "robber" continue so constantly to give me an opportunity to "rob" them? In the first place the people who make such remarks are mistaken. They only *think* they are "robbed," because when they receive their bills the amount seems to them excessive as compared with what they have been accustomed to paying. They only think of the sum total of the bill, and jump to the conclusion that my charges are exorbitant—consequently "robbery." They do not, for a moment, consider that there is as much difference between dentists and dental operations as there is between human faces; that is a lesson for them to learn, and given time and experience they *do* learn it, and in innumerable instances I have had such people come back to me years afterward and penitently admit that my charges were very reasonable. Why? Because they had learned their lesson, that in buying dental services, the important thing to consider was the man and not the price. They had learned that a poor operation was very costly if it was performed for even nothing—while a conscientious and skillful one was cheap, no matter how much was paid for it. Having learned that lesson they became teachers and advisors to their relatives and friends. Confidence in the integrity and ability of their dentist was established, and success in that circle was assured for both parties.

I have always tried to have the courage of my convictions, and I was early convinced that I had two duties to perform. One, and that the first one, to my patient, and secondly, to myself and family. Having done for my patient the very best that my brains and ability would permit, I had the courage to charge an honest and reasonable fee—*and collect it,* and being a pioneer in this community in that method of conducting a dental practice, I found it very hard and slow work, as I was opposed by both the people and neighboring practitioners, who were not accustomed to such definite, business-like methods; but that the method was correct, time has demonstrated fully, for after over thirty years of continuous practice in this city, the educated, refined and wealthy classes of this and surrounding communities seem more anxious than ever to be "robbed."

Of course, all dissatisfied patients, have not returned to me, and probably would not if I lived and practiced till the crack of doom; but what of that! A man has only two hands, and can do only so much work in a day, and if he can maintain without adventitious aids a practice that fills every hour of every working day with a long appointment list ahead; can select from among the applicants those only for whom he wishes to operate, he can be assured that no matter what hasty opinions may be expressed by the unqualified, or what venomous darts may be hurled from unkindly professional sources, the deep and solid foundation laid so many years ago was built upon a rock so firmly that it has been equal to the task of bearing without fear of crumbling the structure of over thirty stories that has been erected upon it, and I doubt not as many more as the physical abilities of the builder will permit.

Dr. George Baker.—I believe there is a right way to use arsenic, and when so used it is a very valuable drug; for instance, I, myself, have had the best results in the use of arsenic when using a very small quantity and allowing it to stay in the tooth about six hours. That is long enough. When I am about to use arsenic I generally tell the patient to come in at nine o'clock in the morning, and return at three in the afternoon, when it is generally possible to extirpate the pulp painlessly. Of course, it is possible to get along without it, but at the same time it does save valuable time. To get along without it, we can take the dental engine and destroy the pulp, under a general or local anesthetic, especially when the pulp is inflamed and arsenic has no more effect on it than water upon a duck's back. If we take 1-50 of a grain of arsenic and an equal quantity of cocaine and mix it with trikresol and make a comparatively thick paste, say of the constituency of butter, then apply a minute quantity of it directly to the pulp and leave it for six hours, it will be possible to extirpate the pulp painlessly. After the pulp is extirpated it should not be left open with a dressing in it that will allow infection to enter the pulp canal, but the canal and tooth should be filled at once. There is very small chance of any trouble following. I have over one hundred recorded cases of this kind, and every one has been successful with this method.

The question was discussed: Does not the effect of arsenic extend beyond the apical foramen? If it does we get as a first symptom periodontitis. If there is no inflammation in the peri-apical tissues, it is apparent there is no arsenic effect beyond the foramen. I think it is very necessary to bear in mind that the use of arsenic is always attended with some danger. Recently, I saw a case where arsenic was applied and it had leaked out into the tissues. The result was necrosis. I removed the sequestrum between the bicuspids, and there was no further trouble; so it is a dangerous thing if it gets out of the cavity and into the tissues. There is bound to be trouble, and we can never tell how far that trouble may extend.

Dr. Brigham.—The dental profession is under great obligation to Dr. Smith of Philadelphia for what he has brought forward. Now, this is not original with him. Many years ago Dr. Riggs practised the same thing, but he never put it on the systematized basis that Dr. Smith has. I happened to be one of the few whose names were sent in to Dr. Smith to attend the clinic which he was to give, but unfortunately for me, Dr. Holly Smith and a few others got ahead of me, and I was left out.

A man who has never practised prophylaxis in a systematic way has no idea of what he can do or how quickly he can restore an unwholesome mouth to a wholesome one. He can change the condition of the saliva from a thick to a good, healthy, thin saliva.

It is a difficult matter to remove these deposits from the necks of the teeth. These deposits we have in two different forms. We have this dark deposit that gathers around the neck in a ridge that is easily removed, but we have another that forms in a thin layer and goes down deep under the gum. This is difficult to remove, and might pass observation, but if you will once break through that deposit you will find the enemy of the teeth, and you will find that you can scale it off. As Dr. Smith says, many times it cannot be removed at one sitting, but it can be removed after some time. You can remove the greater part of it with the scalers, but you can never get it smooth until you polish it with pumice stone and the orange stick. I have made the remark many times that it was like cleaning rust from a piece of steel. You can get off a great deal of it by scraping, but you cannot get it smooth until you polish it.

Dr. Smith spoke of looking at our business in the light of the patient. That is what we should all do. A merchant looks at his business as a customer does, and so strives to please the customer. We should look at our work and make a study of it from the patient's side, once in a while wonder if we would want such and such a thing done for ourselves. I never allow myself to do for a patient what I would not want or would not be willing to have done for myself. That is one of the reasons why I do not grind down many teeth to put on a gold crown.

Patients, in the matter of prophylaxis, will readily co-operate with you, and are willing to come as often as you can take care of them, if you will tell them what you want of them.

As to the use of the rubber dam, I could not get along without it at all. I can do simple operations without the dam, but they have to be very simple. I can apply the rubber dam and get through with the operation much quicker and know that the result is to be quite satisfactory.

As to the use of matrices, I use them continuously, but I do not use those that are prepared and on the market. I make, like Dr. Ainsworth, one for each case. I can cut out the matrix and put it in place and hold it there in a few minutes. I wedge it up with cotton. That may seem like a soft wedge, but you can do it for plastic filling, and it holds it in place very well. I have filled many medium-sized cavities with gold with the matrix held up with cotton only. You can hold it up there as tightly as you wish to.

As to the use of arsenic, I do not see how I could get along without it. Of course, we do not know its limitations, but what has never troubled us ought not to bother us much.

As to the use of porcelain, with me that would be indispensable today. It is as useful to me as any of the fillings which we have. We have four or five filling materials and every one has its place. Cohesive gold I use in the anterior teeth where I think it best. I do not feel sure of putting in a thin cavity with porcelain, and I never do it where it comes to great stress. On corners I use a pin baked in the porcelain. This is of great advantage. You may think it is a very difficult matter to bake a pin in porcelain, but it is just as easy to bake a pin in as it is to bake it without. There may be certain corners which you hardly have a chance to put the pin in, although the porcelain would look the nicest I believe that many of those teeth could be restored to greater usefulness by using gold. They may equal what I saw today, some corners built out in gold in frail teeth some six years ago, and they looked just the same today as they did the day they were done.

Dr. Geo. C. Ainsworth.—I am glad to hear Dr. Smith speak in commendation of the matrix, for outside of Dr. Louis Jack, I believe Boston is pioneer in its use. It certainly has been dosed out to you generously at my hands. Could I have known twenty-five years ago what I know today regarding the use of the matrix, I could have saved my patients and myself many a tedious hour. I would go further than Dr. Smith and say that by its use we save one-third the time, are able to utilize the good qualities of both non-cohesive and cohesive gold, produce far better insurance against future decay, fill teeth with gold that otherwise would be impracticable, and in every way find it an advantage. The men who speak of the matrix as "a snare and a delusion" are not masters of the detail in its use. Ready-made matrices have never found much favor with me. I prefer to make the matrix myself for each case. It takes but two or three minutes to make a matrix, using what is known as cold-rolled French steel, which has a temper peculiar to itself. This is readily burnished on a large piece of Faber's erasion rubber into the desired shape, and may be held against the teeth in a variety of ways. Where you have two compound approximal cavities facing each other in bicuspids or molars, the matrix is readily held in position for one by filling the other with pink gutta-percha; again it may be ligated on or held by a Perry separator. Many times a bit of orange wood at the cervical wall is of advantage.

One advantage in the matrix made by yourself is that you throw it away when you got through with it, thus avoiding the care and sterilizing. It is very simply made, and to my mind it fills the bill better than any of the matrices I have ever seen. Some of the contrivances are very far from what they should be, and do not aid but really prevent doing contour work in the full sense of the word.

As to the matter of filling front teeth with gold or porcelain, there are certain conditions under which I am as yet unable to do as satisfactory work with porcelain as with gold, still I find that I am using more and more porcelain inlays as I surmount the difficulties that are presented in its use. I have been rather slow in taking up porcelain work, waiting for others to get the road well trodden down.

